# The effect of ovarian injection of autologous platelet rich plasma in patients with poor ovarian responder: a systematic review and meta-analysis

**DOI:** 10.3389/fendo.2023.1292168

**Published:** 2023-12-12

**Authors:** Xuanling Li, Huicong Liu, Guangyao Lin, Lianwei Xu

**Affiliations:** Department of Gynecology, Longhua Hospital, Shanghai University of Traditional Chinese Medicine, Shanghai, China

**Keywords:** intra-ovarian injection, autologous platelet rich plasma, poor ovarian responder, review, meta-analysis

## Abstract

**Objective:**

To evaluate the effects of ovarian injection of autologous platelet rich plasma (aPRP) on patients with poor ovarian responder (POR) based on the existing clinical evidence.

**Methods:**

According to systematic review and meta-analysis, we comprehensively searched nine databases established as of September 6, 2023, and evaluated the impact of ovarian PRP infusion on poor ovarian responder. The research results include serum follicle-stimulating hormone(FSH) and anti-Mullerian hormone(AMH) levels, antral Follicle Count(AFC), oocyte number, and embryo number. The Newcastle Ottawa Scale (NOS) was used to evaluate the quality of inclusion in trials.

**Results:**

Add up to 10 studies consisting of 793 participants were included in the meta-analysis. A review of existing evidence showed that intraovarian injection of PRP has significant therapeutic effects in increasing levels of anti-Müllerian hormone (AMH) (SMD=0.44,95% CI [0.07,0.81], p=0.02), antral follicle count (AFC) (MD=1.15,95% CI [0.4,1.90], p=0.003), oocyte count (MD=0.91, 95% CI [0.40, 1.41], p=0.0004), and embryo number (MD=0.78, 95% CI [0.5,1.07], p<0.0001). We compared the relevant data of patients before and after treatment after 2 months of intervention. It can be seen that ovarian injection of PRP treatment for 2 months has better effects in reducing FSH levels, increasing AMH levels, increasing antral follicle count, and increasing the number of oocytes and embryos (p<0.05). When the dose of PRP injected into each ovary was ≥ 4ml, there was also a significant correlation (p<0.05) with improving the number of AFC, oocytes and embryos. Significant heterogeneity existed among the studies.

**Conclusion:**

The pooled results suggest that intra-ovarian injection of PRP can promote ovarian regeneration and improve the reproductive outcomes of patients with ovarian dysfunction. This therapy may have significant clinical potential in improving sex hormone levels, increasing AFC, oocyte count, and embryo count. However, this findings still requires more rigorous and extensive trials worldwide to determine the value of intra-ovarian injection of PRP in POR patients.

**Systematic review registration:**

https://www.crd.york.ac.uk, Identifier CRD42023451232.

## 1 Introduction

POR is a pathological state characterized by poor ovarian response to gonadotropins (Gn) stimulation (1). In women with poor ovarian response (POR), poor response to ovarian stimulation during assisted reproductive techniques such as *in vitro* fertilization (IVF) or intracytoplasmic injection (ICSI) often leads to a decrease in the number of retrieved eggs and pregnancy rate (2). Research has revealed that the incidence of POR is 9-24% (3–5).

Age, anti-Müllerian hormone(AMH), antral follicle count (AFC), and the number of eggs obtained are considered the main indicators for diagnosing POR (1). At present, the clinical treatment of POR includes dehydroepiandrosterone (DHEA) (6), coenzyme Q10 (7), acupuncture and moxibustion (8, 9), platelet rich plasma injection (10–12), etc. Several studies have proclaimed that DHEA supplementation can improve the persistent pregnancy or live birth of POR receiving IVF cycle (13), coenzyme Q10 is conducive to reducing the ROS level in oocytes, inhibiting apoptosis, enhancing mitochondrial function, thus improving ovarian reserve (14), and acupuncture and moxibustion treatment also has significant clinical potential in improving POR, which is manifested in improving hormone level of POR women and repairing ovarian function (9, 15). However, there are obvious differences in acupuncture and moxibustion intervention (16). The efficacy of DHEA is unstable, and most PORs do not respond after application (17). Coenzyme Q10 is vulnerable to multiple factors, and its oral bioavailability is low. At present, its application method, effective dose and safety are still controversial (18, 19).

Platelet rich plasma (PRP) is a concentrate of PRP protein extracted from fresh whole blood, also known as autologous conditioned plasma. It removes red blood cells through centrifugation, thereby exerting anti-inflammatory and regenerative functions (20). In the past two decades, platelet rich plasma has gradually become a widely used treatment method, attracting great attention from medical professionals, mainly due to the potential of PRP in enhancing regeneration processes (21). In multiple clinical studies, PRP has been found to be useful for participating in tissue regeneration and repair in various fields, such as skin diseases (22, 23), osteoarthritis (24), intervertebral disc degeneration(IDD) (25), infertility (26), etc. In addition, autologous platelets are believed to promote the development of isolated human primordial and primary follicles to the pre-antral stage (27). A large quantity of studies have confirmed that intrauterine infusion of PRP is beneficial for follicle maturation in all aspects and has a positive impact on the pregnancy outcomes of patients with unexplained repeated implantation failures (12, 28, 29). However, although there are currently multiple plans for preparing and injecting PRP, there is still no consensus on the optimal plan, and there is still controversy over whether ovarian injection of PRP can improve ovarian function (30, 31). Recent research data suggests that ovarian injection of PRP appears to effectively increase ovarian reserve markers, improve ovarian angiogenesis, follicle formation, menstrual cycle recovery, and ovarian function, contribute to increased egg production (10, 32, 33). Accordingly, we conducted a systematic review and meta-analysis of existing relevant studies, and conducted key evaluations to provide information for clinical practice. The specific objectives of this study are as follows: whether intra-ovarian injection of PRP is effective in improving hormone levels, egg count, etc. in POR patients, and whether it can have a positive impact on assisted reproductive outcomes such as *in vitro* fertilization?

## 2 Materials and methods

This study complied with the preferred reporting items for Systematic reviews and Meta-Analyses (PRISMA) (34) and was registered on PROSPERO (registration number CRD42023451232).

### 2.1 Search strategy

From its inception up to to September 6, 2023, a total of nine databases were retrieved, including 6 English databases: Scopus, Web of Science, EBSCO, X-MOL, PubMed, and Springer, as well as 3 Chinese databases: VIP Information, Wanfang, and China National Knowledge Infrastructure. The search strategy is composed of two constituents: clinical condition (poor ovarian responder) and intervention (platelet rich plasma, autologous platelet rich plasma). Additionally, we carefully evaluated the relevant references of the retrieved research to obtain more potential related articles.

### 2.2 Inclusion and exclusion criteria

Clinical trials that meet the following inclusion criteria would be included: (1) In accordance with the diagnostic criteria of ESHRE and the American Society of Reproductive Medicine (ASRM) POR diagnostic consensus (35) formed in Bologna in 2011, POR is diagnosed by meeting two of the following three characteristics: 1) female age ≥ 40 years old or having other POR risk factors (such as Turner syndrome, history of ovarian surgery, and history of cancer treatment); 2) In the previous assisted reproductive cycle, the ovarian response was relatively low, that is, after receiving conventional ovulation induction protocols, the number of eggs obtained was ≤ 3; 3) Abnormal detection of ovarian reserve function, i.e. AFC<5-7 or AMH<0.5-1.1 ng/ml. (2) Articles investigated the effect of injecting PRP on ameliorating ovarian function in POR patients through phase II clinical trials or retrospective analysis. (3) The intervention measures that meet the conditions are intra-ovarian injection of PRP. (4) There is at least enough data in the study to indicate sex hormone levels or related clinical parameters.

The exclusion criteria were as follows: (1) Less than 10 patients in the study. (2) Research was repetitive publications, reviews, meta-analyses, research protocols, and animal experiments. (3) The research had not been published in English or Chinese.

### 2.3 Data extraction and quality assessment

In accordance with the above qualification criteria, all data is independently extracted using pre-designed tables. Extract research characteristics (author’s surname, publication time, and sample size) from each included clinical trial, as well as relevant results after implementing intervention measures. Whatever disagreements will be resolved by consulting the third observer (L.W.X.). Besides, two reviewers (X.L.L. and H.C.L.) independently evaluated the quality of the included studies (NOS).

### 2.4 Statistical analysis

Stata/MP 17.0 and Review Manager 5.3 software were applied for statistical analysis, while EndNote 20.2 software was used for data management. Summarize continuous data using standardized mean difference (SMD) or mean difference (MD) and 95% confidence interval (CI). Evaluate heterogeneity between studies using I^2^ statistical data. I^2^ ≤ 50% indicates low statistical significance. A fixed effects model should be used. Otherwise, a more suitable random effects model will be employed. Bilateral p ≤ 0.05 indicates a statistically significant difference. So as to compare the effectiveness of different intervention measures in treating POR and explore potential sources of heterogeneity, subgroup analysis was applied. Evaluate the impact of a single study on the overall analysis results through sensitivity analysis, reveal potential heterogeneity and bias, and test the robustness of meta-analysis conclusions.

## 3 Results

### 3.1 Included articles

**Figure 1:**
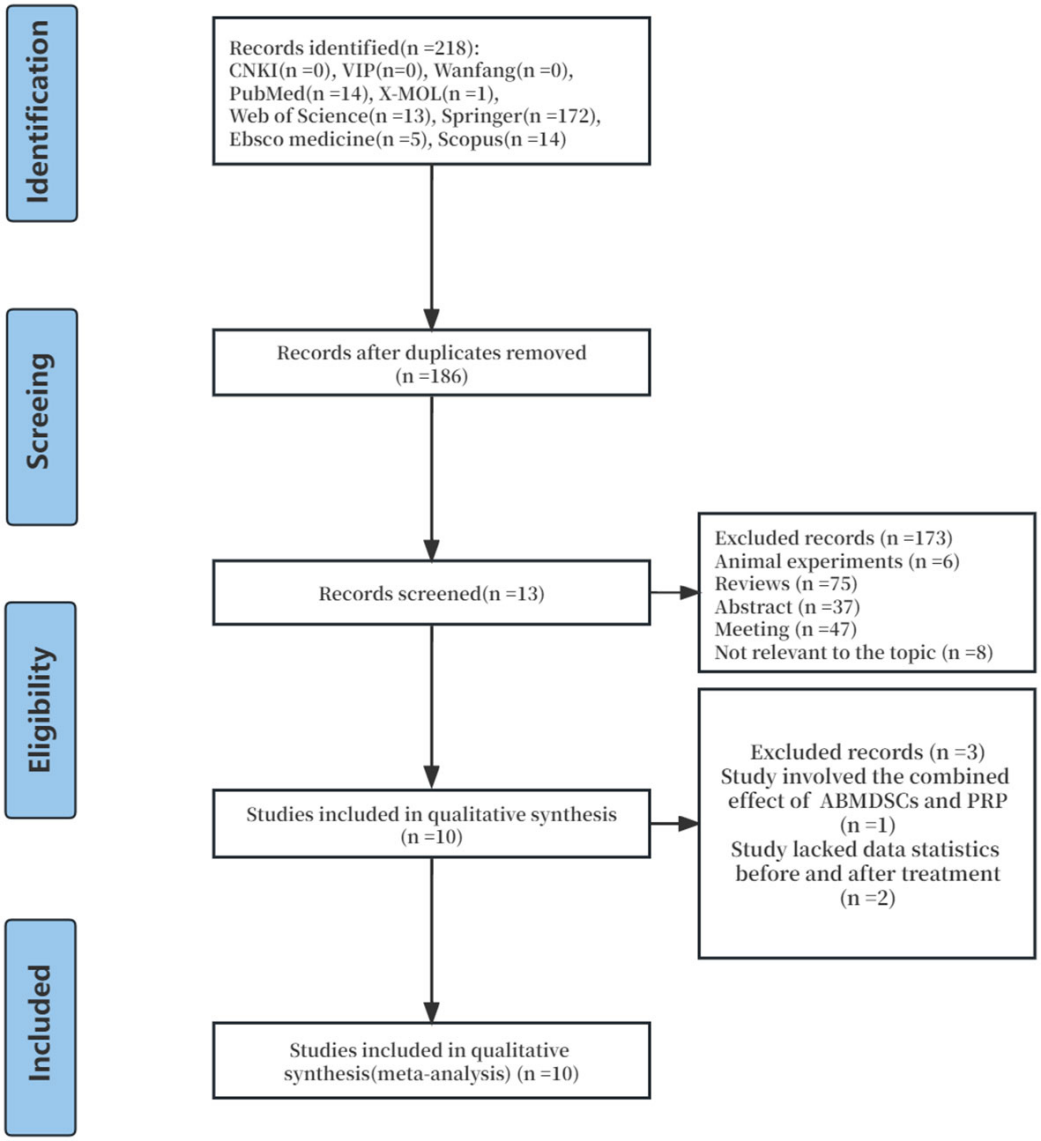
Flow diagram of the selection process.

Through preliminary database search, a total of 218 articles on the clinical efficacy of injecting PRP in the treatment of POR were identified. Out of 218 articles, 32 duplicate articles were excluded, and 173 papers were deleted because they did not conform to the inclusion criteria. Then, we carefully deleted the other three studies as they did not have enough data for analysis or did not accord with the inclusion criteria. Finally, 10 clinical trial studies published from 2019 to 2023 were enrolled in the meta-analysis. The selection process is shown in **Figure 1**.

### 3.2 Study characteristics

**Table 1** summarizes the research characteristics of these clinical trials. Quantitative synthesis was conducted on 10 studies through meta-analysis. The sample size of these studies ranged from 12 to 510, add up to 793 POR patients. Among the 10 trials included, 5 were treated for 2 months (3, 32, 38, 40, 41), 3 for 3 months (37, 42, 43), 1 study for 2-3 months (36), and 1 study for 6 months (39). Three studies injected PRP doses≥4ml per ovary (32, 37, 40), seven studies injected PRP doses <4ml per ovary (3, 36, 38, 39, 41–43), and ten studies used self-control studies (3, 32, 36–43).

**Table 1:** Study characteristics.

	Study	Year	Sample size(n)	Age(year)	BMI	Infertility duration	Intervention	PRP dose	Treatment duration	outcomes	NOS
1	Farimani (36)	2019	12	35.57 ± 3.80	NA	6.50 ± 3.77	PRP	2ml per ovary	2-3 months	①②	7
2	Sfakianoudis (37)	2020	30	38.40 ± 2.01	23.12 ± 2.52	5.83 ± 1.02	aPRP	4ml per ovary	3months	①②③④⑤	7
3	Aflatoonian (3)	2021	17	35.47 ± 4.34	25.68 ± 2.58	4.41 ± 3.18	aPRP	1.5ml	2 months	④⑤	6
4	Farimani (38)	2021	56	40.00 ± 5.00	NA	NA	PRP	2ml/each time	2 months	①④⑤	7
5	Pacu (39)	2021	20	37.4 ± 4.00	NA	NA	PRP	2-4ml	6months	①②③④⑤	6
6	Keikha (40)	2022	12	40.40 ± 3.91	22.59 ± 9.76	2.79 ± 1.72	aPRP	4 ml/right	70days	①②③④⑤	7
7	Cakiroglu (32)	2022	510	40.3 ± 4.0	NA	NA	aPRP	4-8ml	2 months	①②③④⑤	7
8	Parvanov (41)	2022	66	40.5 (34–46)	23.5 (19.0–27.0)	NA	aPRP	1ml per ovary	2months	①②③④⑤	7
9	Tulek (42)	2022	50	38.1 ± 4.4	25 ± 3.4	NA	aPRP	2ml per ovary	3months	①②	6
10	Davari Tanha (43)	2023	20	41.80 ± 1.82	25.85 ± 3.16	9.70 ± 1.89	PRP	3ml per ovary	3months	①④⑤	7

NA, not available; ① Oocyte number; ② Embryo number; ③ Antral follicle count (AFC); ④ Anti Muller hormone (AMH); ⑤ Follicle stimulating hormone (FSH).

### 3.3 Quality evaluation

All studies are self controlled and retrospective cohort studies, and quality evaluation is conducted according to NOS. Among them, 7 items were rated as 7 points. Three studies received 6 points. Although all evaluated studies are of high quality, a common reason for low research quality evaluation scores is the lack of sufficient detail in the outcome evaluation process. **Table 1** shows the NOS scores for each included study.

### 3.4 Outcome measurements

#### 3.4.1 Main research indicators

**Figure 2:**
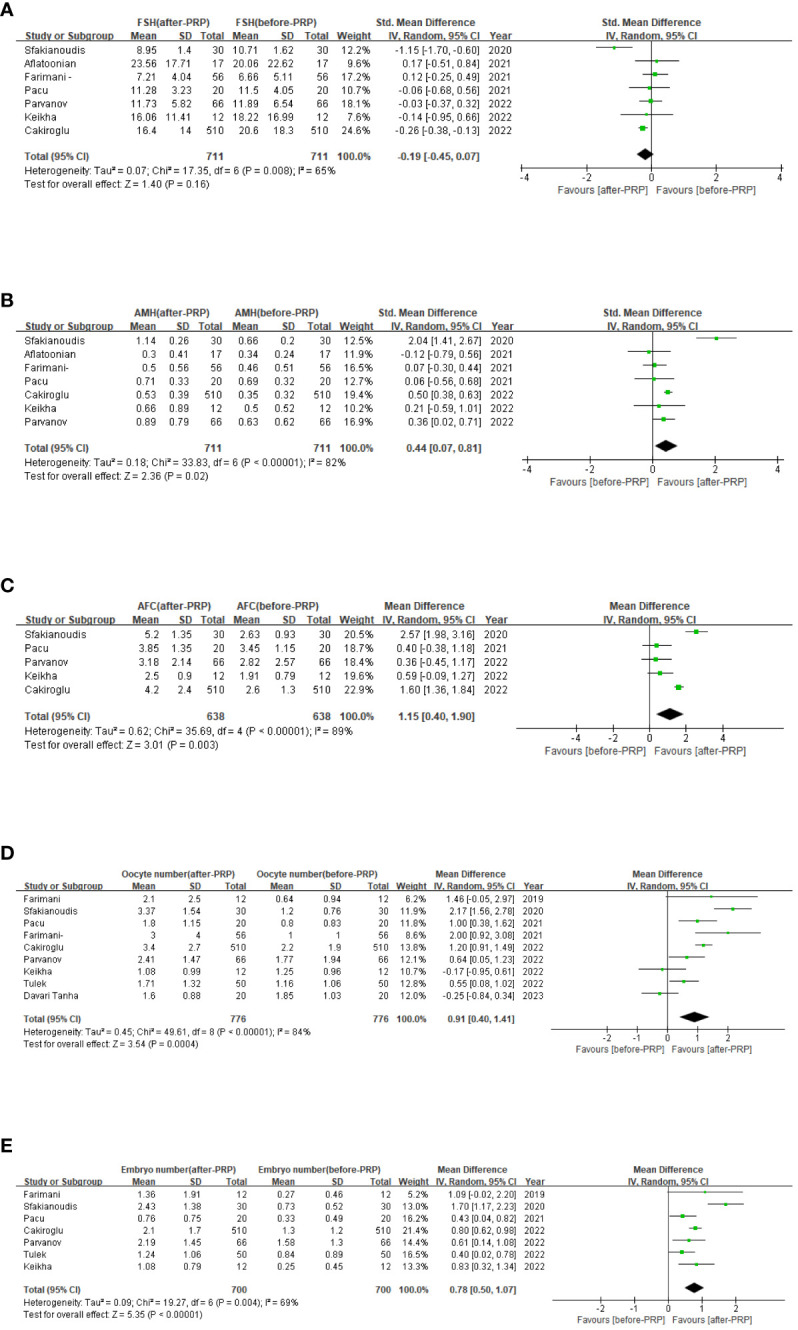
Ovarian injection PRP treatment related forest map, FSH levels **(A)**; AMH levels **(B)**; AFC **(C)**; Oocyte number **(D)**; Embryo number **(E)**.

With regard to hormone levels (**Figure 2**), the summary results showed an increase in AMH levels (SMD=0.44,95% CI [0.07,0.81], I^2^ = 82, p=0.02), while FSH levels (SMD=-0.19,65% CI [-0.45,0.07], p=0.16), did not show significant improvement during the study period. Due to significant heterogeneity in the results of FSH, AMH,a random effects model was used.

**Figure 3:**
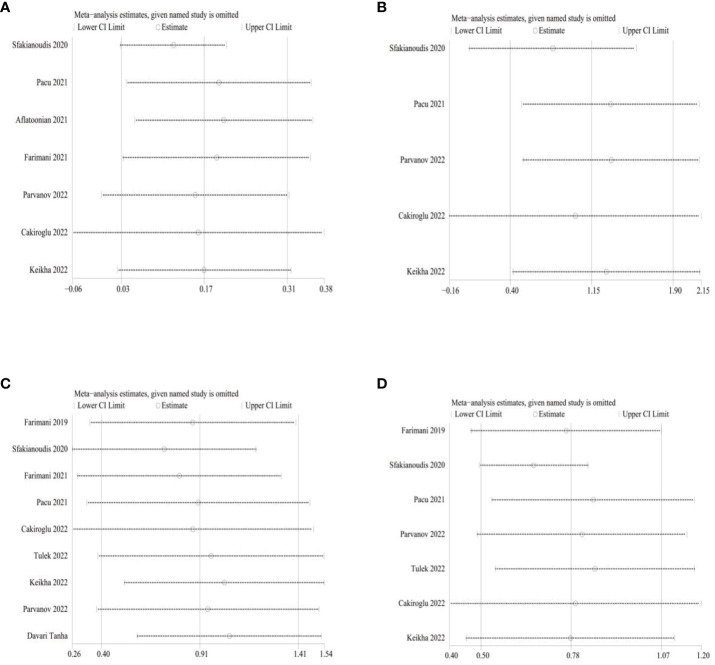
Sensitivity analysis **(A)** for AMH; **(B)** for AFC; **(C)** for Oocyte number; **(D)** for Embryo number.

Amount to 6 studies involving 638 patients were included in a meta-analysis of AFC results. Due to the significant heterogeneity of these studies (I^2^ = 89), we applied a random effects model and the merger results indicated that AFC (MD=1.15,95% CI [0.4, 1.90], I^2^ = 89, p=0.003, **Figure 2**). Nine studies involving 776 patients were included for the analysis of oocyte count results. Due to the significant heterogeneity of these studies (I^2^ = 85), we applied a random effects model and pooled results show the number of oocytes (MD=0.91,95% CI [0.40, 1.41], I^2^ = 84, p=0.0004, **Figure 2**). And 7 studies involving 700 patients were included in a meta-analysis of embryo count results. Owing to the significant heterogeneity of these studies (I^2^ = 69), a random-effect model was applied, and the merged results revealed the number of embryos (MD=0.78,95% CI [0.50, 1.07], I^2^ = 69, p<0.0001, **Figure 2**). Now that the high heterogeneity of each indicator, sensitivity analysis was applied to evaluate the overall impact of individual studies on the main outcome indicators AMH, AFC, oocyte number, and embryo number in order to evaluate the stability of outcomes. The results indicated that after excluding the literature one by one, significant statistical differences did not change, indicating that the meta results were stable (**Figure 3**).

#### 3.4.2 Secondary research indicators

In addition, MII oocytes obtained from POR patients receiving IVF or ICSI were measured in 5 studies, and 2PN embryos were recorded in 3 studies. The combined results indicated that intra-ovarian injection of PRP may statistically increase the number of MII oocytes (MD 0.97, [95% CI: 0.39,1.55], I ^2^ = 76%, p=0.001) and the number of 2PN embryos (MD 0.93, [95% CI: 0.37,1.49], I ^2^ = 87%, p=0.001). Three studies have documented the average dose of gonadotropins, the duration of gonadotropin stimulation and the level of E2 on the day of HCG administration, although the average dose of gonadotropins used after PRP treatment was not statistically significant, the duration of gonadotropin stimulation(MD -1.03, [95% CI: -1.42,-0.63], I ^2^ = 14%, p<0.00001) slightly decreased. On the other hand, the level of E2 (SMD 1.19, [95% CI: 0.25,2.14], I ^2^ = 88%, p=0.01) increased compared to when PRP was not used on the day of HCG administration, which is considered statistically significant. Moreover, 3 studies recorded Cleavage Stage Embryos and cancellation rate, the comprehensive results indicated that intra-ovarian injection of PRP to a certain extent Increased number of cleavage stage embryos(MD 0.93, [95% CI: 0.55,1.30], I ^2^ = 79%, p<0.00001) and diminished the cancellation rate (OR 0.36, [95% CI: 0.21,0.63], I^2^ = 0%, p=0.003) of POR patients. Using sensitivity analysis, there is no single study that affects the merged results. All the above results are tabulated in **Table 2**.

**Table 2:** Summary of clinical results from forest maps.

Clinical outcomes	Studies(n)	Cases(n)	OR/SMD/MD 95% CI	P	I^2^ (%)	Model
MII Oocytes Obtained	5	178	0.97 [0.39, 1.55]	0.001	76	Random
E2 (hCG Trigger)	3	100	1.19 [0.25, 2.14]	0.01	88	Random
2PN Embryos Obtained	3	590	0.93 [0.37, 1.49]	0.001	87	Random
Cleavage Stage Embryos	3	590	0.93 [0.55, 1.30]	<0.00001	79	Random
Duration of stimulation (days)	3	100	-1.03 [-1.42, -0.63]	<0.00001	14	Fixed
Gonadotropin Dose (IU)	3	100	-0.12 [-0.58, 0.34]	0.62	60	Random
Cancellation rate	3	93	0.36 [0.21, 0.63]	0.0003	0	Fixed

#### 3.4.3 Subgroup analysis

Overall results show that in subgroup analysis for different intervention measures, after 2 months of ovarian injection of PRP, serum AMH, FSH levels, AFC, oocyte count, and embryo count increased more significantly (p<0.05), in subgroup analysis for different intervention measures.

Additionally, high-dose injection of PRP (≥ 4ml) into each ovary was beneficial for increasing AFC, oocyte count, and embryo count (p<0.05). Low dose injection of PRP (<4ml) into each ovary is also a significant regulator of FSH, oocyte count, and embryo count. But the data shows that injecting high-dose PRP into each ovary seems to have an advantage in improving the effect.The subgroup analysis results of the correlation between different intervention measures and hormone levels, AFC, oocyte number, and embryo number were tabulated in the **Table 3**.

**Table 3:** Subgroup analysis of the correlation between intra-ovarian injection of PRP and various indicators.

Type of Intervention	Studies(n)	Cases(n)	OR/SMD/MD 95% CI	P	I^2^ (%)	Model
**2 months intervention time**						
AMH	7	711	0.55 [0.17, 0.92]	0.004	82	Random
FSH	7	711	-0.34 [-0.67, -0.01]	0.05	78	Random
AFC	5	638	1.50 [0.76, 2.24]	<0.00001	88	Random
Oocyte number	6	676	0.94 [0.46, 1.43]	0.0001	68	Random
Embryo number	5	650	0.72 [0.56, 0.88]	<0.00001	5	Random
**2-3 months intervention (PRP dose≥4)**						
AMH	3	552	0.93 [-0.09, 1.94]	0.07	91	Random
FSH	3	552	-0.50 [-1.08, 0.08]	0.09	77	Random
AFC	3	552	1.64 [0.71, 2.58]	0.0006	91	Random
Oocyte number	3	552	1.10 [0.07, 2.13]	0.04	91	Random
Embryo number	3	552	1.08 [0.55, 1.60]	<0.00001	80	Random
**2-3 months intervention (PRP dose<4)**						
FSH	4	159	0.26 [-0.13, 0.66]	0.20	63	Random
AMH	4	159	-0.24 [-0.80, 0.32]	0.39	81	Random
AFC	2	86	1.26 [-0.54, 3.07]	0.17	88	Random
Oocyte number	6	224	0.75 [0.22, 1.29]	0.005	71	Random
Embryo number	4	148	0.49 [0.26, 0.72]	<0.00001	0	Random

## 4 Discussion

As early as the 1980s, when ART was used for ovarian stimulation, a certain proportion of patients showed poor ovarian response, with a relatively small count of retrieved oocytes (44). Due to the small count of participants and the heterogeneity between POR defined trials, there is insufficient evidence to determine that using any specific intervention to improve the treatment outcomes of patients with adverse reactions is reasonable. Therefore, POR remains one of the challenges faced by infertility experts (45).

Broer et al. conducted two meta-analyses and found that AMH and AFC had the highest accuracy in predicting ovarian overreaction and poor ovarian response (46, 47). Currently, serum AMH measurements have been added to the factor list, including age, BMI, duration of low fertility, basal FSH, and AFC. The algorithm that includes FSH, AFC, and AMH measurements is called Ovarian Reserve Test (ORT) (48). In theory, AFC can reflect the remaining ovarian follicle pool, and relevant studies have shown that the original follicle pool and ovarian reserve in the ovary are related to the number of growing sinus follicles (49). Hosseini L et al. found that supplementing the culture medium with PRP can better support the vitality and growth of early human antral follicles *in vitro* (27). In a study conducted in 2018, Sills et al. (50) found that intra-ovarian injection of fresh, self activated PRP increased serum anti Muller’s hormone (AMH) levels in patients with ovarian dysfunction, and this effect was related to baseline platelet concentration rather than age or duration of infertility, indicating that granulosa cells are key operators of PRP response.

Biomaterials can be considered as temporary extracellular matrix for stem cells and play an important role in proliferation, differentiation, and formation of new tissues (51). An important component of tissue engineering is growth factors, which are secreted by platelets and are one of the most important sources of cell proliferation and targeted cell differentiation, including PDGF and TGF β,VEGF, IGF-1, FGF, and EGF (52). Therefore, platelet concentrates such as platelet rich blood (PRP), platelet rich protein (PRF), and growth factor rich plasma (PRGF) have been widely used in tissue engineering (53). Research has shown that PRF can better promote the healing of soft and hard tissues (52). The application of PRGF in bone regeneration is increasing (53). Platelet rich plasma (PRP) is currently one of the most commonly used regenerative reagents in clinical practice, which can release growth factors and proteins that have beneficial effects on wound healing and regeneration processes (54). A recent study conducted by S.Herraiz et al. (55) on the improvement of ovarian reserve and reproductive outcomes in women with reduced ovarian reserve by injecting plasma rich in growth factors into the ovaries showed that injecting PRGF into the ovaries helps reactivate follicular growth, promote *in vitro* fertilization cycle initiation, and embryo generation. However, there is still a lack of relevant clinical research on intraovarian injection of PRGF. Intraovarian PRP injection has been used in different case series and cohort studies, and has achieved encouraging results.

In 1974, Kohler and Lipton discovered in their research on the physiology of fibroblasts that platelets as growth stimulants may have significant implications (56). Further research has shown that platelets are a source of growth factors that stimulate fibroblast activity (57). Fausto Cremonesi et al. found that applying autologous platelet rich plasma (PRP) to the left ovary of cows before superovulation helped increase the number of follicles after gonadotropin therapy, thereby promoting the recovery of donor cow embryos (58). Later, in another study by Fausto Cremonesi et al., they included 12 cows with ovarian dysfunction. After 2 months of treatment, they found that administering PRP to the ovaries improved ovarian function and believed that this may be achieved by reducing follicular atresia or restoring dormant oocytes, thereby restoring fertility (59). In addition, Lange Conglio et al. treated ovarian slices on slides with PRP and found that at 48 hours of cultivation, 40-60% of follicular wall cells expressed significant and widespread Ki-67 positivity, and after using PRP, E2 and AMH levels were significantly higher, revealing that PRP can stimulate granulosa cell proliferation and play a role in combating inflammation (60). Studies in various fields have found that PRP can promote cell proliferation and migration (61, 62), angiogenesis (63, 64), and reduce inflammatory reactions, oxidative stress, and aging (65, 66). In addition, research has found that PRP appears to function through different signaling pathways, such as AKT/ERK (63, 64), PGC1 α- TFAM (65), Keap1-Nrf2 (66), Wnt/β- Chain proteins (67), TLR4 (68), PI3K/Akt (69), and Akt/Band/Bcl-2 (70). However, there is still a lack of research on the pathways involved in PRP promoting ovarian regeneration and improving POR, and further research is needed to elucidate its related mechanisms.

Kakudo et al. suggest that one probable interpretation for the impact of PRP on the ovaries is that it acts in a way that promotes angiogenesis through cytokines released by platelets, such as VEGF (71). Ono et al. (72) suggest that the possible interpretation for the positive effect of PRP on the ovary is through sphingosine 1-phosphate (S1P), and there is proof that S1P can promote follicular maturation, possibly by increasing the expression of CCN2, which is a connective tissue growth factor that drives follicular maturation (73). Urtz et al. also proposed a similar view (74). Moreover, in another subsequent study, it was found that PRP can improve the *in vitro* growth and viability of pre antral follicles separated from human ovaries after death, which supports the view that PRP may assist in ovarian regeneration by supporting the development of existing primordial follicles (27). Therefore, it can be seen that intraovarian injection of PRP will be an ideal treatment option for improving POR, and it is a low-cost, simple, and minimally invasive application. Currently, there is no risk of adverse reactions related to drug or foreign body surgery treatment (54). However, it should be noted that like any surgery, PRP also has contraindications and is not recommended for patients with coagulation disorders (75). Other known contraindications include breastfeeding, pregnancy, cancer diagnosis or active infection, and the use of chronic non-steroidal anti-inflammatory drugs (76).

As far as we know, our article is the first meta-analysis to estimate the effect of intra-ovarian injection of PRP in repairing ovarian function in POR patients. In this study, we included 10 self controlled trials involving 793 patients to investigate the correlation between intra-ovarian injection of PRP and improvement in POR. There is evidence to suggest that intra-ovarian injection of PRP therapy can elevate AMH levels, increase AFC, oocyte and embryo numbers. In order to offer more convincing evidence, reduce heterogeneity, and explore potential factors that may make an impact on the clinical efficacy of intra-ovarian injection of PRP, we conducted a subgroup analysis of different intervention types. The results revealed that after 2 months of ovarian injection of PRP, the data of various indicators showed better results. According to the research results composed by the included PACU et al. (39), after 6 months of injection of PRP, Multiple indicators have returned to similar levels as before the PRP plan, so we believe that intervention after injecting PRP for two months is more effective in assisting reproductive outcomes. In addition, we found that when the dose of PRP injected into each ovary was ≥ 4ml, it had significant advantages in increasing AFC, oocyte count, and embryo number compared to when the dose injected into each follicle was<4ml. Although there was no statistically significant difference in subgroup analysis of the improvement effect of PRP injection doses<4ml and ≥ 4ml on patients’ AMH (P>0.05), the overall results showed that patients’ AMH did indeed improve after treatment. Therefore, we believe that the possible reason is that the subgroup analysis reduced the sample size, resulting in insufficient statistical efficiency, which cannot sufficiently reflect the value of PRP, and is not due to the lack of clinical efficacy of intra-ovarian injection of PRP.

While the clinical pregnancy rate and live birth rate of patients are considered the main outcome indicators that need to be observed in POR patients (1), only one study we included (42) reported relevant data before and after treatment. The results of this study showed that intra-ovarian injection of PRP seemed to have a positive impact on POR assisted reproductive outcomes. However, we haven’t been able to comprehensively explore the correlation between intra-ovarian injection of PRP and pregnancy outcomes, which may be an intrinsical flaw in our meta-analysis. Additionally, we have discussed and analyzed the potential factors leading to severe heterogeneity, and our views are as follows: firstly, all included studies are single center experiments; Therefore, there are significant differences in the preparation of PRP and the dosage adjuvant regimen for injection among different studies. Secondly, the follow-up time and examination techniques of POR patients included in the study after receiving intravenous injection of PRP treatment are inconsistent, which may also lead to heterogeneity. We have adopted strict inclusion criteria and conducted extensive literature searches in order to ensure the quality of the source literature. Although the results of this meta-analysis support the benefits of intravenous injection of PRP in improving POR from a clinical perspective, it should be considered that these findings stem from 10 highly heterogeneous clinical studies. We can only make a weak suggestion to inject PRP into the ovaries as part of improving POR. Further investigation requires more high-quality clinical research validation, and current evidence needs to be strengthened or refuted.

## 5 Conclusion

These findings indicate that injecting at least 4ml of PRP into the ovary as an intervention has good clinical potential in increasing AMH levels, AFC, oocyte number, and embryo number in POR patients. Therefore, intra-ovarian injection of PRP can be recommended for improving POR.

## Data availability statement

The original contributions presented in the study are included in the article. Further inquiries can be directed to the corresponding author.

## Author contributions

XL: Conceptualization, Data curation, Formal analysis, Investigation, Methodology, Software, Supervision, Validation, Writing – original draft, Writing – review & editing. HL: Conceptualization, Data curation, Formal analysis, Investigation, Methodology, Software. LX: Conceptualization, Funding acquisition, Investigation, Methodology, Project administration, Resources, Supervision, Validation, Visualization, Writing – review & editing. LG: Conceptualization, Data curation, Investigation, Methodology, Software, Supervision, Writing – review & editing.
